# Clinical usefulness of urinary biomarkers for early prediction of acute kidney injury in patients undergoing transaortic valve implantation

**DOI:** 10.1038/s41598-023-46015-0

**Published:** 2023-10-30

**Authors:** Yumi Obata, Atsuko Kamijo-Ikemori, Sachi Shimmi, Soichiro Inoue

**Affiliations:** 1 Department of Anesthesiology, St. Marianna University School of Medicine, 2-16-1 Sugao, Miyamae-ku, Kawasaki, Kanagawa 216-8511 Japan; 2 Department of Anatomy, St. Marianna University School of Medicine, Kawasaki, Kanagawa Japan

**Keywords:** Valvular disease, Acute kidney injury

## Abstract

This study aimed to reveal the clinical usefulness of urinary biomarkers for the early prediction of AKI onset after transcatheter aortic valve implantation (TAVI) (n = 173). In this study, 22 (12.7%) patients had AKI, of which 21 had mild AKI and 1 had moderate AKI. Higher levels of urinary liver-type fatty acid binding protein (L-FABP), [tissue inhibitor of metalloproteinases-2] × [insulin-like growth factor-binding protein 7], clusterin and urinary albumin before, after and 4 h after TAVI were associated with AKI onset. However, the time point of higher urinary *N*-acetyl-β-d-glucosaminidase levels related to AKI onset was only before TAVI. No significant differences were found in the area under the receiver-operator characteristic curves (AUC) for predicting AKI onset between urinary biomarkers before TAVI. After TAVI, the AUC (0.81) of urinary albumin was significantly higher than those of any other urinary biomarkers. The sensitivity (0.86) in urinary albumin after TAVI and specificity (0.98) in urinary L-FABP before TAVI were the highest among urinary biomarkers. In conclusion, urinary biomarkers may be clinically useful for early differentiation of patients with a higher or lower risk for AKI onset or early prediction of post-TAVI onset of AKI.

## Introduction

Untreated symptomatic aortic stenosis (AS) is associated with poor prognosis and has been conventionally treated by open cardiac surgery for aortic valve replacement. Since 2002, transcatheter aortic valve implantation (TAVI) has been suggested as a less invasive procedure in patients at high risk for operative complications or death, owing to an advanced age, deteriorated functions of the heart, lung and kidney or presence of cerebrovascular disease^1^. TAVI improves the circulatory dynamics via the reduction of AS and showed similar mortality to open surgery^2^.

However, acute kidney disease (AKI) is easily induced after TAVI in the high risk group and is reported to be related to the aggravation of post-TAVI mortality^3,4^. Post-TAVI AKI is caused by a combination of factors such as peri-procedural hypotension, decreased intravascular volume and direct nephrotoxicity of the contrast medium and specialised treatments focused on post-TAVI AKI have not been established. Therefore, at present, early diagnosis of post-TAVI AKI or early differentiation of patients at higher risk for AKI onset is performed using clinically useful biomarkers, leading to the commencement of rapid prophylactic strategies that prevent the post-TAVI onset of AKI (e.g. maintenance of adequate blood pressure, avoidance of volume depletion or minimisation of the contrast volume).

The Valve Academic Research Consortium-2 (VARC-2) criteria using serum creatinine (SCr) or urinary volume is recommended for the diagnosis of post-TAVI AKI^5^. However, SCr has a slower response after AKI onset and has lower sensitivity regarding the accurate reflection of the glomerular filtration rate in patients with poor renal function or low muscle mass^6–8^. In addition, urinary volume during or after TAVI is influenced by the infusion volume or use of diuretics. Therefore, SCr and urinary volume are considered not suitable markers for early diagnosis or early differentiation of patients at higher risk of post-TAVI AKI.

Recently, several urinary new biomarkers of kidney injury, such as urinary liver-type fatty acid binding protein (L-FABP), [tissue inhibitor of metalloproteinases-2] × [insulin-like growth factor-binding protein 7] ([TIMP-2] × [IGFBP7]), or clusterin, have been noticed for early diagnosis of AKI. However, the clinical utility of urinary biomarkers in post-TAVI AKI has not been investigated enough. There were relatively few clinical studies to reveal the usefulness of urinary clusterin in AKI. In this study, we aimed to examine the clinical usefulness of their new biomarkers in addition to conventional markers, such as urinary albumin and urinary N-acetyl-β-D-glucosaminidase (NAG), in post-TAVI AKI.

## Results

### Comparison of characteristics between patients with and without AKI onset

To define AKI onset according to VARC-2 criteria, SCr was measured before operation (pre-op), immediately after operation (post-op), 4 h after operation (4-h post-op) and on post-operative days (PODs) 1, 2, 3, 4, 5, 6 and 7. AKI onset was observed postoperatively in 22 (12.7%) of the 173 patients enrolled in the study. Regarding AKI severity based on the VARC-2 criteria, 21 patients had mild AKI (stage 1) and 1 had moderate AKI (stage 2).

Increased body weight (*P* = 0.006), decreased ejection fraction (*P* = 0.011), higher coincidence of diabetes mellitus (*P* = 0.049) and chronic kidney disease (CKD)(*P* < 0.001) were significantly observed in patients with AKI (AKI group) compared with those without AKI (non-AKI group), whereas no significant differences in sex, age, body mass index, aortic mean gradient, coincidence of hypertension, chronic obstructive pulmonary disease and concomitant medications of β-blockers, calcium antagonists, angiotensin converted enzyme inhibitors/angiotensin II receptor blockers, statins, diuretics and non-steroidal anti-inflammatory drugs were found between AKI and non-AKI groups (Table 1).

**Table 1:** Patient characteristics.

	AKI group (n = 22)	Non–AKI group (n = 151)	*P* value
Sex (M/F)	13/9	53/98	0.059
Age (years)	84 [80–88]	84 [80–86]	0.78
Body weight (kg)	58 [50–66]	51 [43–60]	0.006*
BMI (kg/m^2^)	24 [21–26]	22 [20–5]	0.066
Ejection fraction (%)	60 [52–67]	67 [59–72]	0.011*
Mean gradient (mmHg)	39 [26–46]	38 [25–55]	0.591
Diabetes mellitus (%)	8 (36)	26 (17)	0.049*
Hypertension (%)	22 (100)	124 (82)	0.131
Ischemic heart disease (%)	7 (32)	50 (33)	1.000
COPD (%)	8 (36)	36 (24)	0.425
Chronic kidney disease (%)	21 (95)	90 (60)	< 0.001*
Stage 2	1 (5)	58 (38)	
Stage 3a	6 (27)	48 (32)	1.000
Stage 3b	5 (23)	33 (22)	0.361
Stage 4	6 (27)	9 (6)	1.000
Stage 5	4 (18)	0 (0)	0.82
Concomitant medications, n (%)			
β-blocker	7 (32)	51 (34)	1.000
Ca antagonist	12 (55)	66 (44)	0.361
ACE inhibitor/ARB	10 (45)	67 (44)	1.000
Statin	9 (41)	59 (39)	0.82
Diuretic	7 (32)	40 (26)	0.432
NSAIDs	0 (0)	1 (0.7)	1.000
Preoperative urinary L-FABP (μg/gCr)	5.5 [2.4–60.9]	2.8 [1.3–4.9]	0.005*
Preoperative urinary [TIMP-2] × [IGFBP7]([ng/ml]^2^/1000/gCr)	86.42 [38.01–293.27]	43.48 [13.75–99.44]	0.007*
Preoperative urinary Clusterin (μg/gCr)	94.6 [33.4–1415.2]	16.9 [7.1–78.4]	< 0.001*
Preoperative urinary NAG (mg/gCr)	6.8 [4.0–12.6]	3.5 [2.0–6.2]	0.005*
Preoperative urinary albumin (mg/gCr)	56 [21–2288]	19 [11–44]	< 0.001*
Preoperative sCr (mg/dl)	1.31 [1.05–2.95]	0.84 [0.68–1.05]	< 0.001*
Preoperative eGFR (ml/min)	34 [17–47]	60 [44–66]	< 0.001*

Data are expressed as median [interquartile range] or number (%).

*AKI* acute kidney injury, *BMI* body mass index, *COPD* chronic obstructive pulmonary disease, *ACE inhibitor* angiotensin converted enzyme inhibitor, *ARB* angiotensin II receptor blocker, *NSAIDs* non-steroidal anti-inflammatory drugs, *L-FABP* liver-type fatty acid binding protein, *TIMP-2* tissue inhibitor of metalloproteinases-2, *IGFBP7* insulin-like growth factor-binding protein 7, *NAG* *N*-acetyl-β-d-glucosaminidase, *SCr* serum creatinine, *eGFR* estimated glomerular filtration rate. eGFR was calculated according to the Japanese coefficient-modified Chronic Kidney Disease Epidemiology Collaboration equation: eGFR = 194 × (creatinine)^−1.094^ × (age)^−0.287^ × (0.739 if female)^27^.

*P < 0.05.

### Comparison of operative and post-operative data between AKI and non-AKI groups

No significant differences were noted in the kind of expansion (self-expansion or ball-on expansion), balloon aortic valvuloplasty, anaesthesia duration, operative duration, infusion fluid volume, estimated blood loss, urinary output, blood transfusion and albumin administration (Table 2). The volume of the contrast agent was significantly fewer in the AKI group than in the non-AKI group (*P* = 0.002, Table 2). The presence of mechanical ventilation was not significantly different between the two groups (Table 2). The length of hospital stay was longer in the AKI group than in the non-AKI group (*P* < 0.001, Table 2). One patient in the AKI group required renal replacement therapy in more than 7 days after TAVI, and no in-hospital deaths occurred (Table 2).

**Table 2:** Postoperative date and Clinical Outcomes.

	AKI group (n = 22)	Non-AKI group (n = 151)	*P* value
Self expansion/ballon expansion	3/19	26/125	0.824
BAV	0.52 ± 0.69	0.41 ± 0.58	0.563
Duration of anesthesia (minutes)	137 [121–189]	140 [117–179]	0.923
Duration of surgery (minutes)	71 [61–107]	77 [60–102]	0.767
Fluids infusion (ml)	1100 [913–1753]	1350 [1050–1600]	0.583
Estimated blood loss (ml)	16 [10–102]	10 [10–49]	0.352
Urinary output (ml)	200 [138–833]	400 [250–620]	0.115
Contrast agent (ml)	47 [21–105]	84 [66–112]	0.002*
Blood transfusion (ml)	670 [0–1023]	530 [0–960]	0.698
Albumin administration (%)^†^	12 (54.5)	88 (58.3)	1.000
Volume of injected albumin (ml)	250 [0–500]	250 [0–500]	0.763
Operative and postoperative details			
Mechanical ventilation, n (%)	2 (9.0)	2 (1.3)	0.078
Length of hospital stay (days)	23 [13–26]	11 [9–17]	< 0.001*
RRT required upon discharge, n (%)	1 (4.5)	0 (0)	0.126
In-hospital death, n (%)	0 (0)	0 (0)	–

*AKI* acute kidney injury, *BAV* balloon aortic valvuloplasty*, RRT* renal replacement therapy.

*P < 0.05.

^†^Number (%) of patient with albumin administration.

### Changes in urinary biomarkers between the AKI and non-AKI groups

In this study, urinary L-FABP, [TIMP-2] × [IGFBP7], clusterin, NAG and albumin were measured pre-operation, post-operation, 4-h post-operation and PODs 1, 2 and 3. Preoperative levels of urinary L-FABP (*P* = 0.005), [TIMP-2] × [IGFBP7] (*P* = 0.007), clusterin (*P* < 0.001), NAG (*P* = 0.005) and albumin (*P* < 0.001) were significantly higher in the AKI group than in the non-AKI group (Table 1). In addition, decreased renal function was observed in the AKI group (*P* < 0.001, Table 1).

**Figure 1:**
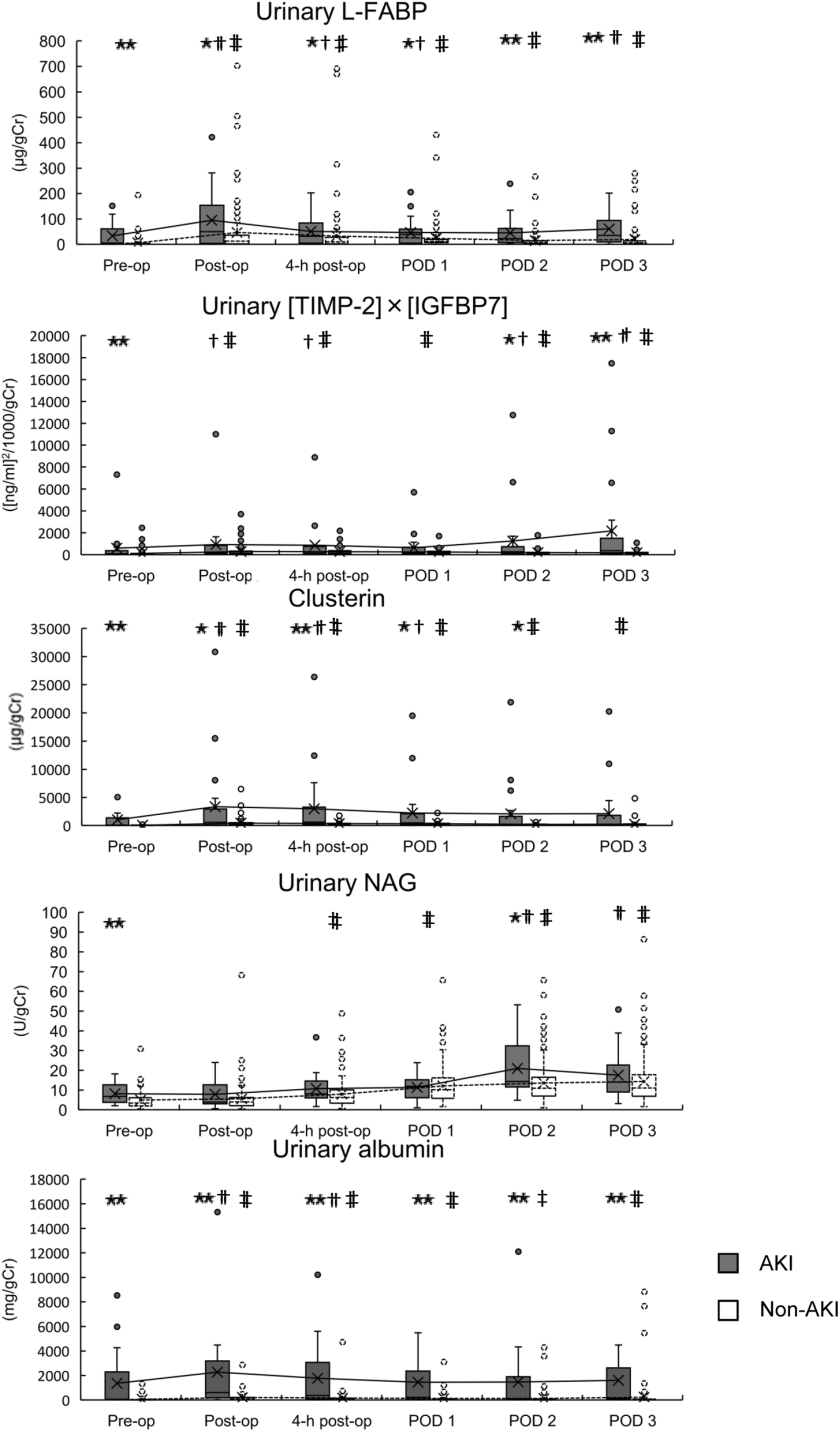
Box and whisker plot showing urinary liver-type fatty acid binding protein (L-FABP), urinary [TIMP-2 (tissue inhibitor of metalloproteinases-2)] × [IGFBP7 (insulin-like growth factor-binding protein 7)], urinary clusterin, urinary neutrophil gelatinase-associated lipocalin and urinary albumin levels measured pre-operation, immediately post-operation, 4-h post-operation and post-operative days (PODs) 1, 2 and 3 in the AKI and non-AKI groups. 4-h post-op, 4 h post-operative value; AKI, acute kidney injury; POD, post-operative day; Post-op, immediate post-operative value; Pre-op, preoperative value. Median and interquartile range values are shown. *P < 0.05, **P < 0.01 vs. level in the non-AKI group at the same time point; ^†^*P* < 0.05, ^††^*P* < 0.01 vs. respective preoperative level in the same (AKI) group; ^‡^*P* < 0.05, ^‡‡^*P* < 0.01 vs. respective preoperative level in the same (non-AKI) group.

Changes in urinary biomarkers in the AKI and non-AKI groups are shown in Fig. 1. In the AKI group, urinary levels of L-FABP significantly increased post-operation (*P* < 0.01), 4-h post-operation (*P* < 0.05) and on PODs 1 (*P* < 0.05) and 3 (*P* < 0.01) compared with those pre-operation (Fig. 1). In the non-AKI group, urinary L-FABP levels significantly increased post-operation, 4-h post-operation and on PODs 1, 2 and 3 compared with those pre-operation (*P* < 0.01, Fig. 1). Urinary L-FABP levels were significantly higher at all measurement points in the AKI group compared with those in the non-AKI group (*P* < 0.05, Fig. 1).

In the AKI group, urinary levels of [TIMP-2] × [IGFBP7] significantly increased post-operation (*P* < 0.05), 4-h post-operation (*P* < 0.05) and on PODs 2 (*P* < 0.05) and 3 (*P* < 0.01) compared with those pre-operation (Fig. 1). In the non-AKI group, urinary [TIMP-2] × [IGFBP7] levels significantly increased post-operation, 4-h post-operation and on PODs 1, 2 and 3 compared with those pre-operation (*P* < 0.01, Fig. 1). Urinary [TIMP-2] × [IGFBP7] levels pre-operation and on PODs 2 and 3 were significantly higher in the AKI group than in the non-AKI group (*P* < 0.05, Fig. 1).

In the AKI group, urinary levels of clusterin significantly increased post-operation (*P* < 0.01), 4-h post-operation (*P* < 0.01) and on POD 1 (*P* < 0.05) compared with those pre-operation (Fig. 1). In the non-AKI group, urinary clusterin levels significantly increased post-operation, 4-h post-operation and on PODs 1, 2 and 3 compared with those pre-operation (*P* < 0.01, Fig. 1). Urinary clusterin levels were significantly higher in the AKI group than in the non-AKI group at all other points, except that on POD 3 (*P* < 0.05, Fig. 1).

In the AKI group, urinary levels of NAG significantly increased on PODs 2 (*P* < 0.01) and 3 (*P* < 0.01) compared with those pre-operation (Fig. 1). In the non-AKI group, urinary NAG levels significantly increased 4-h post-operation and on PODs 1, 2 and 3 compared with those pre-operation (*P* < 0.01, Fig. 1). Urinary NAG levels pre-operation and on POD 2 were significantly higher in the AKI group than in the non-AKI group (*P* < 0.05, Fig. 1).

In the AKI group, urinary levels of albumin significantly increased post-operation (*P* < 0.01) and 4-h post-operation (*P* < 0.01) compared with those pre-operation (Fig. 1). In the non-AKI group, urinary albumin levels significantly increased post-operation (*P* < 0.01), 4-h post-operation (*P* < 0.01) and on PODs 1 (*P* < 0.01), 2 (*P* < 0.05) and 3 (*P* < 0.01) compared with those pre-operation (Fig. 1). Urinary albumin levels were significantly higher in the AKI group than in the non-AKI group at all measurement points (*P* < 0.05, Fig. 1).

### Binomial logistic regression analysis for predicting post-TAVI onset of AKI

In this study, to reveal whether urinary biomarkers at the early phase (pre-operation, post-operation and 4-h post-operation) were associated with AKI onset, binomial logistic regression analysis was performed using ejection fraction, diabetes mellitus and CKD as explanatory variables because these factors were significantly observed in the AKI group compared with the non-AKI group (Table 3).

**Table 3:** Results of binomial logistic regression analyses for AKI in the TAVI.

Variable	Unadjusted (univariate)			Adjusted for DM and CKD			Adjusted for EF and DM and CKD		
	OR	95% CI	*P* value	OR	95% CI	*P* value	OR	95% CI	*P* value
Ejection fraction	0.3	0.117–0.790	0.015*						
Diabetes mellitus	2.77	1.050–7.270	0.039*						
Chronic kidney disease	10.5	2.380–46.70	0.002*						
Urinary L-FABP pre-operation	36.2	8.65–152	< 0.001*	33	5.25–207	< 0.001*	31.0	4.81–200	< 0.001*
Post operation	5.4	2.07–14.1	< 0.001*	3.98	1.46–10.9	0.007*	4.48	1.58–12.7	0.005*
4-h post operation	5.54	2.09–14.7	< 0.001*	3.53	1.26–9.9	0.010*	3.50	1.24–9.93	0.018*
Urinary [TIMP2] × [IGFBP7] pre-operation	5.82	2.14–15.8	< 0.001*	6.9	2.17–22.0	< 0.001*	6.97	2.18–22.3	< 0.001*
Post operation	3.57	1.15–11.1	0.028*	3.74	1.15–12.1	0.028*	3.65	1.12–11.9	0.032*
4-h post operation	3.66	1.30–10.3	0.014*	3.47	1.11–10.8	0.032*	3.41	1.09–10.7	0.035*
Urinary clusterin pre-operation	5.64	2.17–16.4	< 0.001*	5.47	1.88–15.9	0.002*	5.72	1.94–16.8	0.001*
Post operation	9.51	3.40–26.6	< 0.001*	7.46	2.47–22.5	< 0.001*	7.66	2.49–23.6	< 0.001*
4-h post operation	11.3	3.82–33.6	< 0.001*	10.5	3.36–32.8	< 0.001*	10.1	3.22–31.8	< 0.001*
Urinary NAG pre-operation	3.97	1.46–10.8	< 0.001*	2.89	1.01–8.21	0.047*	4.54	1.19–17.3	0.027*
Post-operation	1.04	0.418–2.60	0.928	0.74	0.28–1.97	0.549	0.42	0.04–3.70	0.43
4-h post operation	3.24	1.13–9.31	0.029*	3.24	1.07–9.78	0.038*	0.78	0.27–2.26	0.642
Urinary albumin pre-operation	15.3	4.85–48.4	< 0.001*	9.64	2.65–35.0	< 0.001*	9.71	2.62–36.0	< 0.001*
Post operation	11.6	3.28–41.4	< 0.001*	7.62	2.07–28.0	0.002*	7.91	2.13–29.4	0.002*
4-h post operation	9.14	3.36–24.9	< 0.001*	6.29	2.07–19.1	0.001*	6.21	2.04–18.9	0.001*

*P < 0.05.

*AKI* acute kidney injury, *TAVI* Transcatheter Aortic Valve Implantation, *DM* Diabetes Mellitus, *CKD* Chronic kidney disease, *EF* Ejection fraction, *OR* odds ratio*, CI* confidence interval, *L-FABP* liver-type fatty acid binding protein, *TIMP-2* tissue inhibitor of metalloproteinases-2, *IGFBP7* insulin-like growth factor-binding protein 7, *NAG* *N*-acetyl-β-d-glucosamin.

All urinary biomarkers pre-operation, post-operation and 4-h post-operation, except for urinary NAG post-operation, were significantly associated with AKI onset in the unadjusted analysis and after adjustment for diabetes mellitus and CKD (Table 3). Furthermore, all urinary biomarkers pre-operation, post-operation and 4-h post-operation, except for urinary NAG post-operation and 4-h post-operation, independently predicted AKI onset after further adjustment for ejection fraction (Table 3).

### Receiver-operating characteristic (ROC) curves analysis of urinary biomarkers and SCr for predicting the post-TAVI onset of AKI

Area under the ROC curves (AUCs) of each urinary biomarker for predicting the post-TAVI onset of AKI are shown in Table 4. Although no significant differences in AUC levels were found between urinary biomarkers pre-operation, the AUC level of urinary albumin (0.81) post-operation was significantly higher than any other urinary biomarkers at the same time point (Table 4). Furthermore, post-operation, the AUC level of urinary L-FABP (0.65) was significantly higher than that of urinary NAG (0.41) (Table 4). Moreover, 4-h post-operation, the AUC level of urinary clusterin (0.76) was significantly higher than that of urinary L-FABP (0.63) (Table 4). Each cutoff level of urinary parameters for predicting AKI onset is shown in Table 5. The sensitivity (0.86) of the cutoff value (151.4 mg/g.cr) in urinary albumin post-operation and the specificity (0.98) of the cutoff value (35.3 µg/g.cr) in urinary L-FABP pre-operation were the highest among those of all urinary biomarkers (Table 5).

**Table 4:** AUC Time in the TAVI.

	Pre-op	Post-ope	4-h post-op	POD1	POD2	POD3
Urinary L-FABP	0.68 (0.53–0.83)	0.65^d^ (0.51–0.79)	0.63 (0.48–0.78)	0.64 (0.48–0.80)	0.73 (0.60–0.85)	0.78 (0.66–0.89)
Urinary [TIMP-2] × [IGFBP7]	0.68 (0.56–0.81)	0.62 (0.48–0.75)	0.61 (0.46–0.75)	0.57 (0.43–0.72)	0.66 (0.51–0.80)	0.68 (0.52–0.84)
Urinary clusterin	0.74 (0.61–0.87)	0.67 (0.51–0.83)	0.76^a^ (0.61–0.90)	0.65 (0.49–0.81)	0.64 (0.49–0.79)	0.63 (0.46–0.79)
Urinary NAG	0.71 (0.60–0.82)	0.41 (0.28–0.56)	0.64 (0.52–0.76)	0.51 (0.39–0.64)	0.67 (0.55–0.79)	0.59 (0.47–0.72)
Urinary albumin	0.74 (0.62–0.87)	0.81^a,b,c,d^^(^0.69–0.92)	0.73 (0.59–0.87)	0.71 (0.56–0.85)	0.72 (0.59–0.85)	0.75 (0.63–0.88)

Data are given as AUC (95% confidence interval). ^a^p < 0.05 vs. urinary L-FABP, ^b^p < 0.05 vs. urinary [TIMP-2] × [IGFBP7], ^c^p < 0.05 vs. urinary clusterin, ^d^p < 0.05 vs. urinary NAG.

*AUC* area under the curve, *TAVI* transcatheter aortic valve implantation, *pre-op* preoperation, *after induction* after induction of anesthesia, *post-op* immediate post-operation, *4 h post-op* 4 h after surgery, *POD* post-operative day, *L-FABP* liver-type fatty acid binding protein, *TIMP-2* tissue inhibitor of metalloproteinases-2, *IGFBP7* insulin-like growth factor-binding protein 7, *NAG* *N*-acetyl-β-d-glucosaminidase.

**Table 5:** Biomarkers levels predictive of AKI in the TAVI.

Time point	Cut-off value	Sensitivity	Specificity	PPV	NPV
Urinary L-FABP (μg/gCr)					
Pre-operation	35.3	0.43	0.98	0.75	0.92
Post-operation	45.6	0.62	0.77	0.56	0.92
4 h post-operation	46.3	0.48	0.86	0.35	0.92
Urinary [TIMP-2] × [IGFBP7] ([ng/ml]^2^/1000/gCr)					
Pre-operation	219.79	0.43	0.89	0.35	0.92
Post-operation	91.17	0.81	0.46	0.17	0.94
4 h post-operation	593.23	0.35	0.87	0.27	0.91
Urinary clusterin (μg/gCr)					
Pre-operation	62.7	0.71	0.71	0.25	0.95
Post-operation	1004.9	0.48	0.91	0.44	0.93
4-h post operation	480	0.75	0.79	0.33	0.96
Urinary NAG (mg/gCr)					
Pre-operation	4.4	0.71	0.61	0.21	0.94
Post-operation	3.9	0.52	0.49	0.13	0.88
4-h post operation	6.3	0.76	0.5	0.18	0.94
Urinary albumin (mg/gCr)					
Pre-operation	265.9	0.43	0.95	0.56	0.92
Post-operation	151.4	0.86	0.66	0.26	0.97
4 h post-operation	368.4	0.52	0.89	0.41	0.93

*AKI* acute kidney injury, *TAVI* transcatheter aortic valve implantation, *pre-op* preoperation, *post-op* immediate post-operation, *4 h post-op* 4 h after surgery, *POD* post-operative day, *L-FABP* liver-type fatty acid binding protein, *TIMP-2* tissue inhibitor of metalloproteinases-2, *IGFBP7* insulin-like growth factor-binding protein 7, *NAG* *N*-acetyl-β-d-glucosaminidase.

## Discussion

This study showed that post-TAVI onset of AKI was significantly observed in older patients with decreased ejection fraction, diabetes mellitus or severe CKD. In addition, higher levels of peri-operative urinary L-FABP, [TIMP-2] × [IGFBP7], clusterin, NAG and albumin at the early phase were significantly associated with the post-TAVI onset of AKI. While the AUC levels in each urinary biomarker were not significantly different pre-operation, the AUC in urinary albumin post-operation showed the highest level among all urinary biomarkers measured at the same time point. The cutoff value of urinary albumin post-operation showed the highest sensitivity, whereas that of urinary L-FABP pre-operation showed the highest specificity. Urinary biomarkers may be useful for early differentiation of patients with a higher or lower risk for AKI onset or early prediction of post-TAVI onset of AKI.

The pathophysiology of post-TAVI AKI has not been sufficiently clarified and can be caused by various factors. Previously, the higher volume of the contrast medium injected during TAVI was reported to be a risk factor for contrast-induced nephropathy (CIN)^9^. However, the present study significantly showed a lower volume of the contrast medium in the AKI group than in the non-AKI group. A recent large-scale study did not show a causal relationship between the administration of the contrast medium and AKI^10,11^. On the contrary, preexisting renal insufficiency is the most important risk factor for CIN^12^. In the present study, impaired preoperative renal function was significantly observed in the AKI group compared with the non-AKI group, and the levels of urinary biomarkers that reflect tubular damage were significantly higher in the AKI group than in the non-AKI group before TAVI, showing that the kidneys of patients with AKI may be the most vulnerable to an even lower volume of contrast medium. Furthermore, the levels of urinary L-FABP, which reflects renal ischaemia^13,14^, significantly increased after TAVI than before TAVI in both AKI and non-AKI groups, and levels of urinary L-FABP remained significantly in the AKI group than in the non-AKI group during the peri-operative period of TAVI, suggesting that TAVI caused renal ischaemia and that more severe renal ischaemia may be provoked by TAVI in the kidneys of patients with AKI. A contrast medium induces tubular damage by not only direct toxic effects on tubular cells but also renal ischaemia brought by vasoconstriction^15^. In this study, the contrast medium injected during TAVI may contribute to the induction of post-TAVI AKI in patients with preexisting renal dysfunction and tubular damage.

L-FABP is localised in the cytoplasm of the proximal tubule and is excreted into the urine by ischaemic stress on the tubule before the tissue damage progresses^14^. The clinical usefulness of urinary L-FABP in predicting AKI was reported in various pathophysiology^16–18^, which was supported by the results of the present study. In addition, the specificity of urinary L-FABP before TAVI was the highest among those in all urinary biomarkers. Urinary L-FABP may have potential for the differentiation of patients without post-TAVI onset of AKI in addition to prediction of early post-TAVI onset of AKI.

TIMP-2 and IGFBP7 are related to the induction of G1 cell-cycle arrest^19^. Their expressions are up-regulated in proximal cells by tubular injury; thereafter, they are excreted from the tubules into the urine^19^. Recently, a multicenter study reported the discovery and validation of two novel biomarkers of AKI, i.e. urinary TIMP-2 and IGFBP7^20^, in patients with critical illness^19^. Urinary [TIMP-2] × [IGFBP7] in patients with critical illness after ICU admission was shown to be useful for predicting the development of moderate or severe AKI (KDIGO stages 2–3) within 12 h^19^. In the present study, urinary [TIMP-2] × [IGFBP7] levels significantly increased after TAVI compared with before TAVI in both AKI and non-AKI groups and was significantly higher in the AKI group than in the non-AKI group at the late phase after TAVI. As the G1 cell-cycle arrest in the proximal tubule plays a renal protective role against AKI^21^, the G1 cell-cycle arrest in the tubules was strongly induced in the kidneys of patients with post-TAVI AKI for the prevention of tubular damage. On the contrary, although higher levels of urinary [TIMP-2] × [IGFBP7] after TAVI in addition to those before TAVI were associated with AKI onset, our study did not show a significant difference in urinary [TIMP-2] × [IGFBP7] levels between the AKI and non-AKI groups at the early phase after TAVI. Therefore, urinary [TIMP-2] × [IGFBP7] may be useful in differentiating patients at high risk for AKI before TAVI.

Clusterin is a 5–80 kDa heterodimeric glycoprotein that was reported to regulate apoptosis, lipid transport, cell interactions and complement^22^. Its expression has been reported to be up-regulated in proximal tubular cells depolarised after ischaemia or renal damage and reflects tissue fibrosis^23,24^. Levels of urinary clusterin were significantly higher in the AKI group than in the non-AKI group at both early and late phases, increased significantly after TAVI than before TAVI in both AK and non-AKI groups, and were significantly associated with AKI onset. These results showed the possibility that urinary clusterin may be useful in differentiating patients at high risk of AKI onset before TAVI and predicting early the post-TAVI onset of AKI.

Urinary NAG is a conventional biomarker that reflects proximal tubular damage. In this study, urinary NAG levels significantly increased from 4-h post-operation compared with pre-operation in the non-AKI group but not in the AKI group. On the contrary, significantly higher urinary NAG levels in the AKI group than in the non-AKI group were observed pre-operation but not at the early phase after TAVI. Furthermore, higher levels of NAG before TAVI were associated with AKI onset but not after TAVI. From these results, urinary NAG before TAVI may be useful in differentiating patients at high risk of AKI onset.

Urinary albumin is widely recognized to reflect tubular injury in addition to glomerular damage^25^. The higher levels of urinary albumin before TAVI were reported to be associated with not only AKI onset but also all-cause death and heart failure readmission and may be noticed for predicting the prognosis after TAVI^26^. This study showed the retention of significantly higher urinary albumin levels in the AKI group than in the non-AKI group during the peri-operative period of TAVI and a significant increase in urinary albumin levels at the early phase after TAVI compared with pre-operation in both AKI and non-AKI groups. Urinary albumin levels pre-operation and early phase after TAVI were significantly associated with AKI onset. Furthermore, regarding the time point of immediately after TAVI, the AUC of urinary albumin for predicting AKI onset was the highest among AUCs of all urinary biomarkers. Tubular injury causes the impairment of peri-tubular capillary, which leads to glomerular damage that is located upstream of the peri-tubular capillary. The procedure of TAVI is considered to provoke tubular and glomerular damage. As urinary excretion of albumin is increased by glomerular and tubular damage, urinary albumin may be a prominent marker for predicting AKI onset. This study indicated that compared with other biomarkers, measuring urinary albumin immediately after TAVI is more useful for predicting AKI onset peri-operatively.

The present study showed that the sensitivity (0.86) of the cut-off value (151.4 mg/g.cr) in urinary albumin post-operation and the specificity (0.98) of the cut-off value (35.3 µg/g.cr) in urinary L-FABP pre-operation were the highest among those of all urinary biomarkers. Although this point is needed to be reconfirmed in multicenter studies, measurement of urinary L-FABP before the procedure of TAVI may be useful for discrimination of patients without post-TAVI onset of AKI, whereas measurement of urinary albumin after TAVI may be superior for early predicting the post-TAVI onset of AKI.

We considered that urinary biomarkers which reflect the pathophysiology of post-TAVI AKI may be useful for early prediction of AKI due to TAVI. Contrast medium, which induces proximal tubular injury via both direct toxic actions and renal ischemia, is an important cause for post-TAVI AKI as described above. Therefore, we selected urinary NAG which is increased by construction injury of proximal tubules, and both urinary L-FABP and clusterin which reflect proximal tubular ischemia. In addition, as renal ischemia induces cell cycle arrest and both TIMP-2 and IGFBP7 are related to the cell cycle arrest, urinary [TIMP-2] × [IGFBP7] was measured. Furthermore, clinical impact of urinary albumin on the prognosis after TAVI was recently reported^26^ and urinary albumin was added. On the other hand, both KIM-1 and NGAL have been widely studied and are well-known tubular biomarkers. However, the relationship between urinary KIM-1 and renal ischemia was not clear. Urinary NGAL was reported to be increased by inflammation^27^. We did not determine whether increased urinary NGAL levels after TAVI was due to AKI or infection brought by procedure of TAVI. Thus, urinary KIM-1 and NGAL were not evaluated in the present study.

This study has some critical limitations. First, this was a single-centre study, and the number of patients with AKI was small, which lead to the limited number of variables included in the logistic regression analysis. Second, significant differences were found in the prevalence of CKD, each urinary biomarker level and renal function before TAVI between the AKI and non-AKI groups. The movements of each urinary biomarker after TAVI may be influenced by the presence of CKD or renal dysfunction before TAVI. Third, as albumin administration during the operation of TAVI was performed in the present study, there was a possibility that the administered albumin had an effect on urinary albumin levels. However, urinary albumin levels in the AKI group at all time points were not significantly different between the patients with and the without the albumin administration (Supplementary Fig. S1). Finally, the severity of AKI in nearly all patients with AKI was mild, and the clinical utility of urinary biomarkers for predicting the onset of moderate or severe AKI was not investigated.

In conclusion, urinary biomarkers measured in this study may be clinically useful for early differentiation of patients with a higher or lower risk for AKI onset or early prediction of post-TAVI onset of AKI. In the future, a multicenter study involving more severe AKI cases due to TAVI is needed to reveal the clinical utility and superiority of each urinary biomarker.

## Methods

The study protocol was approved by the Institutional Review Board of St. Marianna University School of Medicine (No. 3049) and registered in the UMIN Clinical Data Registry (ID 000020262). Written informed consent was obtained from all the enrolled patients. The research related to human use has been complied with all the relevant national regulations, institutional policies and in accordance the tenets of the Helsinki Declaration, and has been approved by the authors' institutional review board or equivalent committee.

### Study design

This prospective study was conducted in a university hospital. As contrast medium is one of causes which induces post-TAVI AKI, the sample size calculation was based on the previous study which showed that urinary L-FABP before injection of the contrast medium was useful for early predicting the contrast medium-induced nephropathy (CIN)^28^. The mean difference of urinary L-FABP before the injection of contrast medium between the CIN and non-CIN groups was 11.1 with a larger standard deviation of 12.8. The sample size calculation (α = 5%, power of 80%) resulted in 14 patients in the AKI group and 56 patients in the non-AKI group. In the present study, consecutive patients scheduled for TAVI (n = 173) between December 2016 and June 2020 were enrolled. Each patient’s surgeon chose between balloon expansion and self-expansion by considering the type of the aortic valve, in the absence of any concern regarding the patient’s tolerance for study procedures. Patients undergoing dialysis or requiring emergency operation were excluded from the study.

All patients who underwent TAVI received either a balloon-expandable Edwards SAPIEN transcatheter heart valve (Edwards Lifesciences, Irvine, CA, USA) or a self-expandable Medtronic Core Valve (Medtronic Incorporation, Minneapolis, MN, USA) for clinical indications. Procedures were performed under general anesthesia with endotracheal intubation and insert transesophageal echocardiography for circulation management. TAVI devices were delivered through the femoral approach.

### TAVI AKI study protocol

#### Anesthesia

No premedication was given, and general anesthesia was induced with remifentanil and propofol. Tracheal intubation was facilitated with rocuronium, general anesthesia was maintained with sevoflurane in an air–oxygen mixture, and remifentanil, and noradrenaline was infused continuously during the operation in all patients.

If needed, nicorandil and/or carperitide were infused continuously during the operation in some groups. For transfusion, colloidal fluid, Ringer’s acetate, maltose Ringer, concentrated red blood cell fluid, fresh-frozen plasma, and albumin were used.

### Sample collection

Urine samples (10 mL) were obtained preoperatively on admission (pre-op), immediately after returning to the ward after the operation (post-op), 4 h after returning to the ward (4-h post-op), and POD 1, 2, and 3, respectively, for the measurement of urinary L-FABP, [TIMP-2] × [IGFBP7], clusterin, NAG, and albumin. Urine samples were centrifuged at 1000×*g* for 5 min at 4 °C and stored at − 80 °C until analysis. In addition, serum samples were obtained preoperatively on admission, immediately after returning to the ward after the operation, and on PODs 1, 2, 3, 4, 5, 6, and 7 for the measurement of SCr.

### Clinical monitoring of patients with TAVI

The timing for the diagnosis of AKI was extended from 72 h to 7 days. During the first 48-h postoperative period, patients were monitored for AKI, which was defined according to the VARC-2 criteria^3^. The staging of postoperative AKI was made as follows: stage 1, an increase in SCr to 150%–199% (1.5–1.99 × increase compared with baseline), increase of ≥ 0.3 mg/dL (≥ 26.4 mmol/L), or urine output < 0.5 mL/kg/h for 6–12 h; stage 2, an increase in SCr to 200%–299% (2.0–2.99 × increase compared with baseline) or urine output < 0.5 mL/kg/h for 12–24 h; stage 3, induction of renal replacement therapy.

At the start of this study, the estimated glomerular filtration rate (eGFR) was also monitored, which was calculated according to the Japanese-coefficient modified Chronic Kidney Disease Epidemiology Collaboration equation^29^:

$$ {\text{eGFR}}\, = \,{194}\, \times \,\left( {{\text{creatinine}}} \right)^{{ - {1}.0{94}}} \, \times \,\left( {{\text{age}}} \right)^{{ - 0.{287}}} \, \times \,\left( {0.{\text{739 if female}}} \right). $$

### Assay of urinary L-FABP, urinary [TIMP-2] × [IGFBP7], urinary clusterin, urinary NAG, urinary albumin, and SCr

Urinary L-FABP levels were determined by latex-enhanced immunoturbidimetric assay with anti-human L-FABP mouse monoclonal antibodies (Sekisui Medical, Tokyo, Japan)^30^. Urinary [TIMP-2] × [IGFBP7] levels were determined by enzyme-linked immunosorbent assay (ELISA) with the use of the Human TIMP-2 ELISA kit (R&D System, MN, USA) and Human IGFBP7 ELISA kit (Boster Bio, Pleasanton, USA). Urinary clusterin levels were determined by enzyme-linked immunosorbent assay (ELISA) using the Human Clusterin Quantikine ELISA kit (R&D Systems, Minneapolis, MN, USA). Urinary NAG and albumin levels were measured by immunonephelometry. SCr was measured by an enzymatic method.

### Statistical analyses

Study variables are expressed as median values (interquartile range [IQR]). Between-group (AKI group vs. non-AKI group) differences were analyzed by the Mann–Whitney U test or the chi-square test, as appropriate. After the Friedman test, differences among each group were identified using Dunn’s test. Univariate analysis was performed using the characteristics showing a significant difference between the AKI and non-AKI groups and urinary biomarkers pre-operation, post-operation, and 4-h post-operation. Following the univariate analysis, a multivariate logistic regression analysis in each urinary biomarker was performed, with the addition of ejection fraction and presence of diabetes mellitus or CKD by a forward selection method. ROCs were plotted to identify AUCs and cutoff laboratory values for predicting AKI onset.

All statistical analyses were performed with IBM SPSS Statistics version 21.0 (IBM, Tokyo, Japan), Stat Flex version 6 (Artec, Osaka, Japan), and EZR version 1.3 (Saitama Medical Center, Jichi Medical University, Saitama, Japan)^31^, which is a graphical user interface for R (The R Foundation for Statistical Computing, Vienna, Austria).* P* < 0.05 was considered significant for all analyses.

### Supplementary Information

Supplementary Figure S1.

## Data Availability

The data used to support the findings of this study are included within the article.
