# Supplementary material for: Clinical usefulness of urinary biomarkers for early prediction of acute kidney injury in patients undergoing transaortic valve implantation
Source: Sci Rep. 2023 Oct 30;13:18569. doi: 10.1038/s41598-023-46015-0 (PMC10616062; doi:10.1038/s41598-023-46015-0)
Supplement: Supplementary file 1 — Supplementary Figure S1. [file 41598_2023_46015_MOESM1_ESM.pdf]

Clinical usefulness of urinary biomarkers for early prediction of acute kidney injury in patients undergoing transaortic valve implantation

Short Title: Urinary biomarkers and post-TAVI AKI

Yumi Obata*[1], Atsuko Kamijo-Ikemori[2], Sachi Shimmi[1], Soichiro Inoue[1]

[1]Department of Anesthesiology, St. Marianna University School of Medicine, Kawasaki, Kanagawa, Japan.
[2]Department of Anatomy, St. Marianna University School of Medicine, Kawasaki, Kanagawa, Japan.

***Corresponding author:** Yumi Obata, M.D., Ph D.,
Department of Anesthesiology, St. Marianna University School of Medicine
2-16-1 Sugao, Miyamae-Ku, Kawasaki 216-8511, Japan.
Tel: +81-44-977-8111 ext. 3452; Fax: +81-44-977-8430
E-mail: y2obata@marianna-u.ac.jp

Additional Figure

Supplementary Figure S1

Influence on urinary albumin levels due to albumin administration

The patients in the AKI group were divided into two groups based on the presence or absence of albumin administration and the changes in urinary albumin levels were examined during the perioperative period of TAVI. As a result, urinary albumin levels at all time points were not significantly different between the two groups (Figure S1).

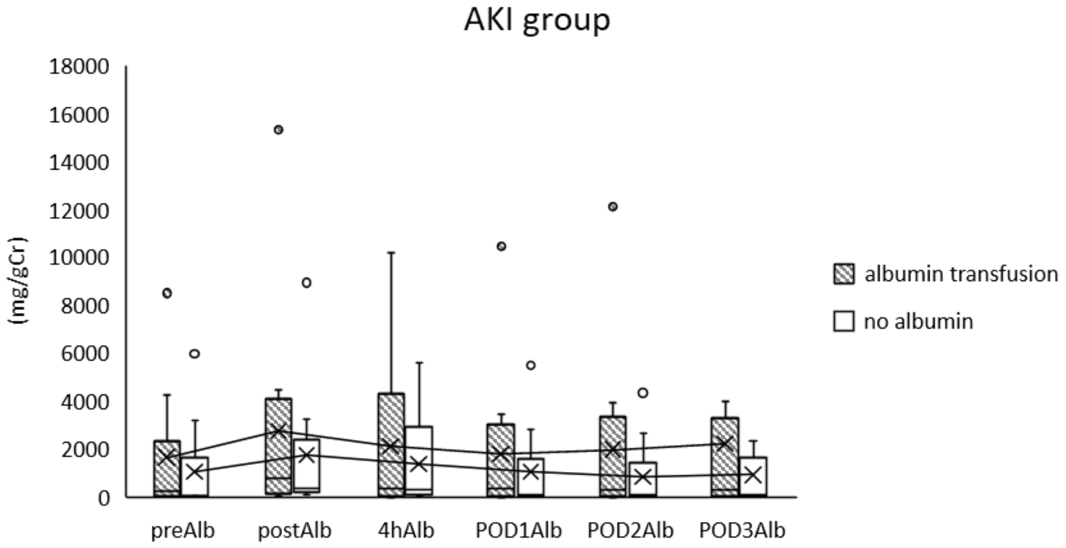

Figure S1 legend

Box-and-whisker diagram of the effect of albumin administration on urinary albumin levels in the AKI group. AKI, acute kidney injury; pre Alb, preoperative albumin value; Post Alb, immediate post-operative albumin value; 4-h Alb, 4 h post-operative albumin value; POD1Alb, post-operative day1 albumin value; POD2Alb, post-operative day2 albumin value; POD3Alb, post-operative day3 albumin value; Median and interquartile range values are shown.
